# The role of pharmacists in enhancing epilepsy care: a systematic review of community and outpatient interventions

**DOI:** 10.1080/20523211.2025.2487046

**Published:** 2025-04-10

**Authors:** Michael Petrides, Aliki Peletidi, Evangelia Nena, Theodoros Constantinidis, Christos Kontogiorgis

**Affiliations:** a Pharmacy Programme, Department of Health Sciences, School of Life and Health Sciences, University of Nicosia, Nicosia, Cyprus; b Laboratory of Hygiene and Environmental Protection, Medical School, Democritus University of Thrace, Alexandroupolis, Greece; c Bioactive Molecules Research Center, School of Life and Health Sciences, University of Nicosia, Nicosia, Cyprus

**Keywords:** Patient safety, pharmacoepidemiology, systematic review, epilepsy care, public health, pharmacist-led interventions and services

## Abstract

**Background:**

Approximately 50 million individuals across the globe are impacted by epilepsy, leading to fear, discrimination, psychiatric issues, high costs, and social stigma. Proper diagnosis and treatment could allow up to 70% of those affected to live seizure-free. Community pharmacists have significant potential to actively participate in epilepsy patient care, beyond merely dispensing medications. The objective of this study was to systematically review and assess the roles of pharmacists in epilepsy care, focusing on pharmacist-led interventions and services for patients with epilepsy.

**Methods:**

Following PRISMA 2020 guidelines, the review included cross-sectional, retrospective cohort, and qualitative/quantitative studies on pharmacist-led epilepsy interventions in community and outpatient settings. Searches were conducted in Scopus, PubMed Central, and Science Direct for studies published through the end of 2023. Two evaluators independently reviewed and chose studies, and the data was analysed using Microsoft Excel®. Quality assessment was performed using the MMAT tool.

**Results:**

Five eligible studies were included, covering 457 participants. Studies originated from the USA (*n* = 3), Netherlands (*n* = 1), and Palestine (*n* = 1). They evaluated pharmacist-led interventions in epilepsy, including medication adherence, quality of life, and pharmacist’s integration in epilepsy care.

**Conclusion:**

This review underscores the possible contributions of pharmacists in epilepsy care, stressing the importance of pharmacist-led interventions to enhance medication adherence and the quality of life for individuals with epilepsy. Future research should evaluate the effectiveness and cost-effectiveness of these services, including disease management and patient education. Increasing awareness among pharmacists and patients about pharmacists’ contributions is crucial for improving epilepsy care.

## Background

Epilepsy is among the oldest recorded diseases in the world, dating back to 2000 BCE (Bone & Dein, 2021; Kaculini et al., 2021). It is a prevalent noncommunicable neurological disorder, affects individuals across all age groups globally, and it is associated with fear, misunderstanding, discrimination, psychiatric comorbidity (Tellez-Zenteno et al., 2007), high economic costs (Begley Charles & Beghi, 2002) and social stigma (Fiest et al., 2014), affecting the quality of life for patients with epilepsy (PWE) and their families (World Health Organisation, 2024). Around 50 million individuals globally are affected by epilepsy, ranking it as one of the most prevalent neurological disorders. The active epilepsy rate is 4–10 per 1000 people. People with epilepsy face a risk of premature death that is up to three times higher than that of the general population. Proper diagnosis and treatment could allow up to 70% of those affected to live seizure-free (World Health Organisation, 2024). Additionally, epilepsy is identified as the second most burdensome neurological disorder globally when considering disability-adjusted life years (Murray et al., 2012). Nevertheless, significant disparities exist in the treatment of epilepsy globally, especially between high-income and low-income countries and between rural and urban areas, within developing regions such as Africa, Latin America, and Asia where it remains highly stigmatised (Meyer et al., 2010). These gaps are primarily influenced by a range of economic, social, political, and cultural factors (Mbuba et al., 2008).

Globally, the population is growing and aging. A 2015 WHO report (World Health Organisation, 2015) noted that the number of people aged 60 and above is increasing faster than any other age group, projected to more than double from 901 million in 2015 to 2.1 billion by 2050. This shift is partly due to healthcare advancements, including technology, nutrition, and lifestyle improvements, leading to longer life expectancy (Babar et al., 2018). As people age, the risk factors for non-adherence to medication rise, with older adults often managing multiple chronic conditions requiring complex medication regimens (George et al., 2008). Consequently, there is a dire need for pharmacists’ contribution in chronic disease management.

### The potential role of community pharmacists in epilepsy

The reduction in medicine manufacturing within pharmacies, coupled with the emergence of new supply and advisory roles for community pharmacists (CPs), has driven educational reforms over the past five decades (International Pharmaceutical Federation (FIP), 2012). A growing number of CPs are now providing direct patient care as well as chronic disease management interventions, leveraging their extensive training and clinical knowledge in health and wellness, preventive care, and patient education (Bacci et al., 2019; Goode et al., 2019). Medication adherence for chronic conditions can be improved by including CPs within multidisciplinary care teams (de Barra et al., 2018; Giberson et al., 2011).

Currently, published literature indicates that CPs play a significant role in chronic disease management, particularly diabetes, hypertension, and established cardiovascular conditions. Nonetheless, there is a scarcity of published information regarding the role of community pharmacists in the management of epilepsy patients (de Barra et al., 2018). CPs have a lot of opportunities to help treat epilepsy patients outside of just filling prescriptions. These opportunities include patient education, support for adherence, and clarification of potential drug interactions and adverse effects (Koshy, 2012). Furthermore, they provide education and counselling to patients about their condition and monitor individuals with epilepsy to detect emerging health issues and prevent the progression of comorbidities. They often play an essential role in addressing various needs (Koshy, 2012).

The aforementioned are in line with many of the 10 main suggestions of the Pharmaceutical Group of the European Union (PGEU) Vision for Community Pharmacy 2030 (PGEU, 2019), that can be properly adapted to PWE needs:

(a). Boost pharmaceutical services to enhance treatment outcomes and adherence while lowering risks to optimise the advantages of the CPs’ intervention for PWE and healthcare systems; (b). Provide CPs with access to all appropriate patient health data, including a list of all the medications they take; (c). Enable community pharmacists to manage medications, promote health, and educate the public to assist in lessening the overall impact of chronic diseases.

### Aim

This study aimed to systematically review and evaluate the pharmacists’ roles in epilepsy, focusing on pharmacist-led interventions/services for PWE. Additionally, it examined the extent to which the finally selected articles conform to quality standards as per the Mixed Methods Appraisal Tool (MMAT) (Hong, Fàbregues, et al., 2018).

## Methods

This systematic review followed PRISMA 2020 guideline (Page et al., 2021) (Supplementary Material S1). The drafted protocol was agreed by the researchers before conducting the full review (Supplementary Material S2). The review was not included in any available protocol registries (Campbell, Cochrane, PROSPERO).

### Eligibility criteria and study selection

The criteria for selection were determined by the study search design. This systematic review included cross-sectional studies, retrospective cohort studies, qualitative and/or quantitative studies if (1) they referred to any epilepsy intervention/service, which involved pharmacists within a community setting or an outpatient clinic, (2) they included adult epileptic patients only, and (3) full-text articles were available in English. The keywords employed for this review included: ‘epilep*’, ‘community pharmac*’, ‘service*’, ‘intervention*’, ‘implementation*’, ‘consultation*’, ‘care’, ‘compliance’, ‘adherence’, ‘concordance’, ‘Morisky’, ‘MARS’, ‘educat*’, ‘quality of life’, ‘QOLIE’, ‘hospital pharm*’. The exclusion criteria were the following: (1) review studies, (2) studies conducted within a hospital pharmacy setting, (3) studies using children as population of interest, (4) studies where full-text articles were not available and (5) studies not written in English language.

### Information sources and search strategy

The databases Scopus, PubMed Central, and ScienceDirect were systematically searched from their inception until 31 December 2023. The search strategies employed varied based on the specific requirements and functionalities of each search engine. Keywords and, where applicable, medical subject headings (MeSH) were utilised in conjunction with Boolean operators (AND, OR, NOT) to refine the search results. The truncation operator (*) was applied to capture variations of a word (e.g. ‘epilep’ to include ‘epilepsy’, ‘epileptic patients’, etc.), and double quotes (‘’) were used to search for exact phrases, thereby optimising the search strategy. Supplementary Material S3 provides a presentation of the search strategies.

### Selection process

**Figure 1.**
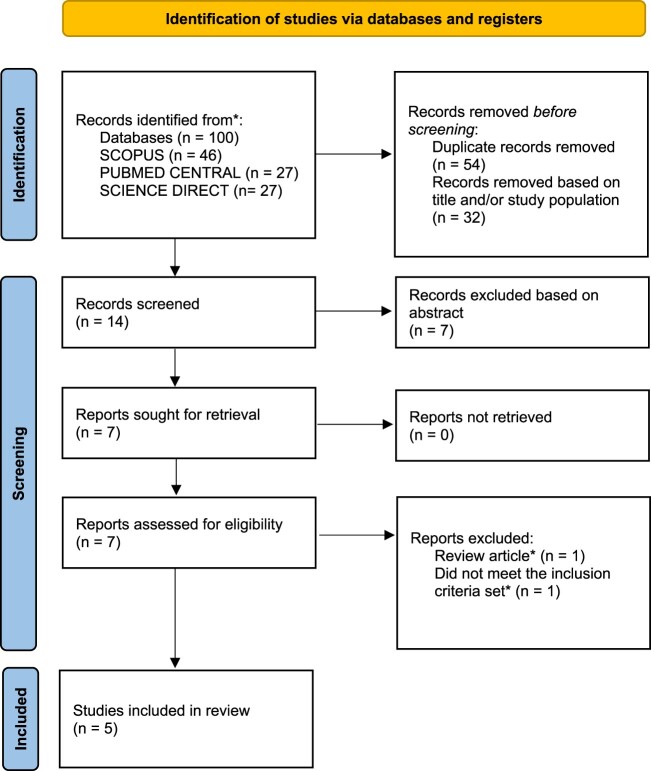
PRISMA flow chart illustrating the process of study selection.

Two assessors, M.P. and A.P., reviewed all eligible studies for proper documentation. Subsequently, the full texts of the chosen articles were independently examined by both assessors. In case of any disagreement, the two assessors resolved it through discussion. All eligible articles were incorporated into Mendeley Reference Manager® for duplicates to be deleted (Figure 1).

### Data collection method and process

All of the finally selected articles were screened independently by each reviewer (all had expertise in pharmacy practice and/or pharmacoepidemiology), who were responsible for carrying out the data extraction. Information on article characteristics was extracted, including the title, publication date, location of publication, primary focus of the article, and study population. Information regarding the scope of the articles was extracted, with an emphasis on the targeted patient populations (adults, patients with epilepsy), practice settings (community pharmacy or outpatient clinic), healthcare professionals (pharmacists), as well as pharmacist-led services provision. Due to the complexity of the interventions under investigation, we sought to classify the included interventions along two dimensions: (A) Pharmacist-led interventions in epilepsy and (Β) Integrating Pharmacists into Epilepsy Care: Developing Patient Services. A summary of findings of the eligible studies visually displays the results of each individual study in Table 1.

**Table 1.** General characteristics of the included studies.

Author & year	Study design & Geographic Area	Main objectives	Population	Methods / tools /measurements	Key findings
McAuley et al. (2015) [20]	Cross-sectional study. USA	Impact of the confounding factors of memory and mood on antiepileptic drug adherence.	100 adult patients with epilepsy (outpatient neurology clinic).	Assessment of: (i) **Subjective memory** (6 memory domain questions from QOLIE-89 questionnaire) (ii) **Objective memory** (Hopkins Verbal Learning Test – Revised) (iii) **Subjective adherence** (Morisky scale – 4 questions) (iv) **Objective adherence** (Medication Possession Ratio – MPR). (v) **Mood** (Neurological Disorders Depression Inventory for Epilepsy)	***Adherence*** *Objective adherence*: 35% (*N* = 35) of the patients were not adherent. *Subjective adherence*: Most patients, whether adherent (ADH) or non-adherent (non-ADH), categorised themselves as having ‘medium’ or ‘high’ adherence. ***Memory*** There were no observed differences between the ADH and non-ADH groups on either the subjective memory scale or the objective memory measure. In the overall sample, neither subjective nor objective memory scores showed a correlation with MPR. However, when comparing correlations between the groups, significant correlations were found between subjective and objective scores within the ADH group (*z* = 2.42, *p* = 0.02), which were absent in the non-ADH group. ***Mood*** Patients in the ADH group exhibited significantly lower mood scores, suggesting a better mood compared to those in the non-ADH group (*p* = 0.04). There was a significant negative correlation between depressive symptoms and subjective memory scores (*r* = −0.52, *p* < 0.0001). However, no significant correlation was identified between depressive symptoms and objective memory scores. ***Seizure activity*** A chi-square analysis indicated a significant interaction between seizure freedom and adherence (*p* = 0.0003). Patients who had been seizure-free for over 12 months were more likely to belong to the ADH group.
Wassenaar et al. (2016) [21]	Community-based retrospective cohort study. Netherlands	Anti-epileptic drug (AED) change patterns and the association between such changes and quality of life (QoL).	248 adult patients with epilepsy.	Assessment of: **Quality of Life** (validated Dutch version of the QOLIE-31).	A total of 77 participants (31%) changed their AED treatment at least once during the 2-year study period, with a median of 1 change (range 1–4). Of these, 43 patients (56%) intensified their treatment, while 34 (44%) reduced it. The reasons for these changes included lack of efficacy (23%), adverse events (AEs) (29%), both lack of efficacy and AEs (18%), concerns about teratogenicity (1%) or unknown reasons (29%). The average QoL score was 77.6 (SD 16.3), adjusted for the number of AEDs used at index date. Patients who changed their treatment had a QoL that was 6.2 points lower (95% CI: −10.4 to −1.9) averaging 73.2, compared to those who did not change their treatment, who had an average score of 79.4. This effect was most pronounced in patients who intensified their treatment, in whom the adjusted QoL score 6.9 points lower (95% CI: −12.6 to −1.2) than those who did not change their treatment. The QoL declined with the number of treatment changes, regardless of whether the change was an intensification or a reduction. Each additional change was associated with a further reduction in QoL (QOLIE-31), with an average decrease of 4.9 points per additional change (95% CI: −7.4 to −2.4, *P*-value < 0.001).
Shawahna (2017) [22]	Prospective observational study using a modified Delphi technique. Palestine	Develop and achieve consensus on a core list of essential knowledge items that community pharmacists (CPs) should be familiar with concerning women with epilepsy.	9 key contacts (3 neurologists, 2 women with epilepsy, 4 CPs), 6 international researchers with interest in women’s issues 30 panelists (22 CPs, 5 hospital pharmacists and 3 academic educators)	(i) Interviews with 9 key contacts (ii) Literature review (iii) Ratings, comments, and suggestions from international researchers (*n* = 6) with interest in women’s issues in epilepsy (iv) Delphi technique with 30 panelists (v) Analytical hierarchy process (AHP) with 10 panelists (10 out of the previous 30).	Final list of 68 knowledge items in 13 categories. Importance weights (%) for each category and each item.
Bacci et al. (2021a) [23]	Qualitative study. USA	Identification of the predisposing, enabling, and reinforcing factors that influence the integration of CPs in population health approaches to epilepsy care.	32 stakeholders, including 5 patients with epilepsy (PWE), 10 caregivers of PWE, 7 epileptologists, 1 neurologist, 1 epilepsy nurse, and 8 CPs in Washington State and Oregon.	Data were gathered through semi-structured interviews with key informants and analysed using a qualitative approach. The PRECEDE–PROCEED Model (PPM) was utilised to guide the collection and analysis of the data, focusing on the design, implementation, and evaluation of health programs.	A range of factors was identified as influencing the integration of community pharmacists into a population health approach to epilepsy care, as recognised by all stakeholder groups: **Predisposing Factors:** These included patient advocacy, adherence to medication, monitoring of medication, and education about medication. **Enabling Factors:** Key elements were a shared vision, a collaborative framework, effective communication, and the attributes of pharmacists, such as their knowledge, experience, and attitude. **Positive Reinforcing Factors:** These comprised a team-based approach, readily accessible support, and adherence to medication. **Negative reinforcing factors:** They included duplicate or conflicting care and limited time and resources.
Zaraa S. et al. (2023) [24]	Design thinking study. USA	Employ design thinking techniques to create a community pharmacist-led intervention for individuals with epilepsy. This intervention should be desirable, feasible, and viable, aiming to improve the quality of epilepsy care.	Interviews were conducted with 5 PWE, 3 caregivers, 6 pharmacists, and 3 pharmacy students, using a semi-structured interview guide. An advisory board consisting of 15 multidisciplinary thought leaders (pharmacists, epileptologists, neurologists, implementation scientists, payers, PWE and caregivers) was established to provide additional input for the final intervention design.	**Prototype development:** Three distinct personas representing individuals with epilepsy were developed to serve as target patients for a community pharmacist intervention. These personas were based on insights from previous research and included: (i) a newly diagnosed patient, (ii) a patient with well-controlled epilepsy, and (iii) a patient with complex needs and poor seizure control. 2. **User feedback:** To involve potential end users in the design process, individual and group interviews were conducted with key informants. This included 5 individuals with epilepsy, 3 caregivers, 6 pharmacists, and 3 pharmacy students. The interviews aimed to gather feedback on the most desirable, feasible, and viable features of the three intervention prototypes. 3. **Final intervention design:** A diverse advisory board consisting of 15 experts from various fields was established to contribute to the final design of the intervention. This board, chosen through purposive sampling, included pharmacists, epileptologists, neurologists, implementation scientists, payers, individuals with epilepsy, and caregivers. The board reviewed the three prototypes and user feedback, providing insights and recommendations on key features of the prototypes, including which features to prioritise. The advisory board and research team worked in small groups to evaluate the feedback and then collectively voted on the significance and relevance of different features. They identified and ranked the four most critical features of the intervention, considering their potential impact on the target population and the feasibility of implementation. The research team then refined and finalised the intervention as a minimum viable product.	The most effective features of a community pharmacist-led intervention for epilepsy were identified as follows: **Patient-pharmacist consultation:** The primary feature is the consultation between the pharmacist and the patient, potentially including the caregiver. This in-person meeting, lasting up to 60 min, focuses on discussing epilepsy care, medications, and quality of life issues. The pharmacist takes a comprehensive approach to understand the patient's health journey, current status, needs, concerns, medication history, and treatment goals. This session also introduces additional pharmacy services, such as automatic prescription refills, medication synchronisation, and adherence tools. The pharmacist can also identify potential drug interactions and assess medication adherence. b. **Care plan development:** The pharmacist collaborates with the patient to develop a customised care plan that addresses the patient's unique health needs, challenges, and objectives. This process includes educating the patient about epilepsy and medication use, discussing possible side effects, and highlighting the importance of following the prescribed treatment regimen. The care plan serves to align the efforts of the entire healthcare team, and the pharmacist may work with other healthcare providers to modify medications and recommend non-pharmacological strategies to enhance overall health outcomes. c. **Regular check-ins:** Frequent check-ins between the patient and pharmacist are crucial. These short meetings, which can occur in-person or through telehealth, enable the pharmacist to track the patient's progress, provide education, and respond to any questions. These interactions foster trust, evaluate the effectiveness of the care plan, and allow for adjustments based on new insights or changing needs. After each session, the care plan is revised to ensure the patient continues to receive suitable care. d. **Care coordination:** Care coordination requires working closely with the patient's healthcare team, including caregivers, to provide holistic and integrated care. The pharmacist plays a key role in the epilepsy care team, collaborating with neurologists, epileptologists, primary care providers, and other healthcare professionals to comprehensively evaluate the patient's needs and medical history. By actively integrating feedback from the care team, the pharmacist refines the care plan to reflect their insights. This collaborative effort ensures that the care plan is adaptable and responsive to the patient's evolving needs, ultimately promoting optimal health outcomes.

*QOLIE* Quality of Life in Epilepsy Inventory, *MPR* Medication Possession Ratio, *ADH* adherent, *non-ADH* non-adherent, *AED* anti-epileptic drug, *QoL* Quality of Life, *AEs* adverse events, *CPs* Community Pharmacists *AHP* analytical hierarchy process, *PWE* patients/persons with epilepsy, *PPM* Precede-Proceed Model

### Quality assessment

Quality assessment of the studies was undertaken by the researchers who worked independently using the English version of the MMAT tool (Mixed Methods Appraisal Tool) (Hong, Fàbregues, et al., 2018), after a pilot practice within the research team in this review. The MMAT is a tool that permits the critical appraisal of the methodological quality of systematic mixed studies reviews, i.e. reviews that include qualitative, quantitative, and mixed methods studies. The MMAT tool includes 25 questions grouped into 5 categories depending on the different study designs, plus 2 screening questions to be answered in all study designs: 1. Qualitative, 2. Quantitative randomised controlled trials, 3. Quantitative nonrandomised, 4. Quantitative descriptive, 5. Mixed methods. For each of the 27 questions the possible answers were three: ‘Yes’, ‘No’, ‘Can’t tell’.

The MMAT quality assessment results are detailed in Table 2. The overall score obtained was 100% for qualitative studies (*n* = 2), 70% for quantitative non-randomised studies (*n* = 2), and 100% for mixed methods studies (*n* = 1).

**Table 2.** Quality assessment according to the MMAT.

Studies	Criteria from the mixed methods appraisal tool															
	1. Qualitative studies					3. Quantitative non-randomised studies					5. Mixed methods					Overall score per study
	1.1	1.2	1.3	1.4	1.5	3.1	3.2	3.3	3.4	3.5	5.1	5.2	5.3	5.4	5.5	
Bacci et al. (2021a)	1	1	1	1	1											100%
Zaraa et al. (2023)	1	1	1	1	1											100%
**Overall score for Qualitative studies (*n* = 2)**	100%	100%	100%	100%	100%											
	**100%**															
McAuley et al. (2015)						0	1	1	1	1						80%
Wassenaar et al. (2016)						0	1	1	0	1						60%
**Overall score for Quantitative non-randomised studies (*n* = 2)**						0%	100%	100%	50%	100%						
						**70%**										
Shawahna (2017)											1	1	1	1	1	100%
**Overall score for Mixed methods studies (*n* = 1)**											100%	100%	100%	100%	100%	
											**100%**					

## Results

### General characteristics of eligible studies

This review article contains five eligible studies (Figure 1 represents the PRISMA flow diagram which illustrates the article evaluation and selection process), published between 2015 and 2023 (Bacci et al., 2021a; McAuley et al., 2015; Shawahna, 2017; Wassenaar et al., 2016; Zaraa et al., 2023). Studies originated from USA (*n* = 3), Netherlands (*n* = 1), and one from Palestine. The general characteristics of the eligible studies are presented in Table 1.

### Literature search and study choice

Out of 100 citations, 54 were duplicates and 32 were ineligible. After assessing abstracts, 7 were excluded, leaving 7 full-text articles to be screened for eligibility. Five articles were finally included because one was found to be a review article, and one did not meet the inclusion criteria set (it was an overview of commonly used antiepileptic drugs – AEDs).

### Features of the selected studies

Following the selection process, this review included five studies, encompassing 457 participants. This review evaluated one cross-sectional study, one retrospective cohort study, two qualitative studies and one mixed-methods study (qualitative and quantitative). Table 1 presents further details on the study features and strategy, as well as the number and characteristics of the participants.

#### Pharmacist-led interventions in epilepsy

Mc Auley et al. examined how memory and mood as confounding factors affect adherence to antiepileptic medication in PWE (100 adult patients, USA, 2015), potentially facing selection and measurement biases due to the nature of the objective tools that were used. Memory and adherence were assessed using both subjective tools (6 memory domain questions from QOLIE-89 and a modified Morisky scale with 4 questions) and objective tools (the Revised Hopkins Verbal Learning Test and the Medication Possession Ratio), whereas mood was evaluated by a subjective tool (the Neurological Disorders Depression Inventory for Epilepsy). Objective adherence data showed that 35% (*n* = 35) of the patients were not adherent (non-ADH group), whereas 65% (*n* = 65) were adherent (ADH group). Additionally, no differences were found between the ADH and non-ADH groups in either the subjective memory scale or the objective measure. In the entire group, there was no correlation between subjective or objective memory scores and MPR. However, when comparing correlations between groups, significant correlations of subjective and objective scores were observed within the ADH group. (*z* = 2.42, *p* = 0.02), which were not present in the non-ADH group. Moreover, patients in the ADH group exhibited significantly lower mood scores, suggesting a better mood, compared to those in the non-ADH group (*p* = 0.04). Finally, a chi-square analysis comparing seizure freedom with adherence showed a significant interaction between the groups. (*p* = 0.0003). Seizure-free patients (those who had been seizure-free for more than 12 months) were more likely to be in the ADH group.

Wassenaar et al. examined patterns of changes in antiepileptic drugs and their relationship with quality of life in PWE (248 adult patients, Netherlands, 2016), which was assessed with QOLIE-31, with risks of attrition bias and uncontrolled confounding variables. At least one drug change was observed in 77 of the participants (31%). Drug change patterns included treatment intensification, substitution, reduction, discontinuation, etc., whereas reasons for changes included lack of efficacy, adverse events (AEs), both lack of efficacy and AEs, concerns of teratogenicity, etc. The average QoL score was 77.6 (SD 16.3), adjusted for the number of AEDs used at index date. Quality of life decreased with the number of treatment changes, irrespective of whether the change involved an intensification or a reduction. Each additional change corresponded to a further decline in QoL (QOLIE-31), with an average decrease of 4.9 points per additional change (95% CI: −7.4 to −2.4, *p*-value < 0.001).

#### Integrating pharmacists into epilepsy care: developing patient services

Shawahna’s study sought to develop and achieve consensus on key knowledge items that community pharmacists should be familiar with regarding women's issues in epilepsy. A modified Delphi technique was used to conduct this consensual study, which may be subject to biases in expert selection and consensus interpretation. Knowledge items were compiled from literature and interviews with nine key contacts to identify what community pharmacists should know about women's issues in epilepsy. Five researchers with an interest in women's issues suggested additional knowledge items and were asked to rate and comment on the collected items. To reach consensus on the core list of knowledge items, two iterative Delphi rounds were conducted with a panel of 30 pharmacists. Ten panellists then ranked these items by importance using the Analytical Hierarchy Process (AHP). A consensus was reached to include 68 knowledge items under 13 categories in the final core list, and each of the items was ranked by its importance by using the Analytical Hierarchy Process (AHP). Using consensual knowledge lists may help standardise the education and training of community pharmacists on women's issues in epilepsy.

Bacci et al. explored the identification of predisposing, enabling, and reinforcing factors that affect the integration of community pharmacists into population health strategies for epilepsy care. This study sought to examine the perceptions of key stakeholders regarding how CPs can most effectively support the chronic care of individuals with epilepsy and contribute to enhancing outcomes for these patients, using qualitative methods that could introduce interviewer and response biases, as well as potential non-representativeness of the sample. The data were collected from semi-structured key informant interviews of the 32 stakeholders that participated in the study (Bacci et al., 2021a, USA) and included 5 PWE, 10 caregivers of PWE, 7 epileptologists, 1 neurologist, 1 epilepsy nurse, and 8 community pharmacists in Washington State and Oregon. The study identified 4 predisposing, 4 enabling, 3 positive reinforcing, and 2 negative reinforcing factors that affect the incorporation of CPs into a population health strategy to epilepsy care. These factors can help direct the development of population health interventions for epilepsy that include community pharmacists.

Zaraa et al. applied design thinking to develop a community pharmacist-led intervention for people with epilepsy, aiming to ensure it is appealing, practical, and sustainable, with possible biases in participant selection and limited generalisability of outcomes. The study employed design thinking and created three patient personas based on previous research: a newly diagnosed PWE, a well-controlled PWE, and a complex PWE with uncontrolled seizures. For each persona, a prototype of the intervention was formulated. Structured interviews with pharmacists, pharmacy students, patients with epilepsy, and caregivers were conducted to gather feedback on the desirability, feasibility, and viability of each prototype's features. Rapid content analysis was used to analyse the interviews. A multidisciplinary advisory group and the research team prioritised the features for inclusion in the final intervention. They identified four features as desirable, feasible, and viable for a pharmacist-led intervention for individuals with epilepsy: (1) pharmacist-patient consultations, (2) development of care plans, (3) regular follow-ups, and (4) coordination of care with other healthcare providers. The study employed design thinking to determine evidence-based features for a community pharmacist intervention to support epilepsy care.

The included studies covered a wide range of community pharmacy interventions, some of which included CPs (McAuley et al., 2015; Wassenaar et al., 2016) (*n* = 2), whereas others involved a multidisciplinary team (Bacci et al., 2021a; Shawahna, 2017; Zaraa et al., 2023) (*n* = 3), including PWE, caregivers of PWE, epileptologists, neurologists, epilepsy nurses, community pharmacists and pharmacy students. The included studies (McAuley et al., 2015; Wassenaar et al., 2016) (*n* = 2) provided details on the tools that were applied during the interventions (e.g. quality of life questionnaires, Morisky questionnaire on drug adherence, etc.). Three exceptions were studies (Bacci et al., 2021a; Shawahna, 2017; Zaraa et al., 2023) that provided details to develop community pharmacist-led epilepsy interventions or factors that affect the integration of CPs in epilepsy care health approaches.

## Discussion

The studies were conducted in a limited number of locations, namely the USA, Netherlands, and Palestine, and focused on specific pharmacy services or activities. They emphasised the impact of mood and memory on adherence to antiepileptic drugs, as well as the patterns of changes in these drugs and their connection to quality of life.

Interestingly, it was observed that there was little attention paid to preventive interventions like patient education and patient training, and disease management interventions including drug monitoring, follow-up, high-risk patient interventions, medication review and/or new medication services and patient behaviour modifications, which are of great importance in patients with epilepsy. Three studies have strongly emphasised the importance of multidisciplinary collaboration that highlights the unique potential of community pharmacists. It is important to recognise their contribution and leverage their expertise to optimise patient outcomes.

### Interpretation

The content and focus of the 2 out of 5 included studies were related to pharmacist-led interventions utilising medication adherence tools (objective and subjective), memory assessment tools (objective and subjective), mood assessment tools, and quality of life tools. While the remaining 3 studies, tried to: (i) identify the factors (predisposing, enabling, and reinforcing) that affect the integration of CPs in population health approaches to epilepsy care, (ii) design an intervention led by CPs for PWE that improves epilepsy care, and (iii) develop a core list of essential knowledge items that CPs should be familiar with concerning women with epilepsy.

Therefore, there is scope to develop, design and evaluate epilepsy patient services through community pharmacies and/or outpatient clinics which will integrate pharmacists in person-centered epilepsy care and management, as part of their role within the multidisciplinary team.

Although all selected studies were published between 2015 and 2023, there is no clear evidence that pharmacists intervene effectively in epilepsy care management. There is an evidence gap regarding the effective and feasible provision of pharmacist-led interventions in the community and/or outpatient clinics, in both screening and disease management. This is in line with Bacci et al. study (Bacci et al., 2021b), which mentioned that there is little published literature on CPs’ role in epilepsy management and suggest further research on the topic.

### Strengths and weaknesses

To the best of our knowledge, this is the first systematic review to evaluate existing published data on the roles of pharmacists in epilepsy, with a focus on pharmacist-led interventions and services for PWE. The review included studies from various locations and employed the validated Mixed Methods Appraisal Tool (MMAT), version 2018, to evaluate the quality of the selected studies. The study assessors (M.P. and A.P.) independently and thoroughly reviewed the texts, discussed their methodologies, and resolved any disagreements encountered during these discussions.

An additional strength of this systematic review is that 3 of the studies included multiple stakeholders (PWE, caregivers, epileptologists, neurologists, community pharmacists, nurses, pharmacy students, etc.) to design community pharmacist-led interventions that improve epilepsy care.

Limitations of the MMAT (version 2018) tool have been previously discussed in the literature. The MMAT tool may be less effective for assessing the quality of studies that do not cleanly fall into the tool's categories. Moreover, some questions may not be applicable to certain types of mixed research methods, which may lead to a lower score for the study. Additionally, the MMAT tool can be time-consuming to use, especially for lengthy or complex research (Hong, Gonzalez-Reyes, et al., 2018). There was also a limited number of studies which covered pharmacists’ contribution to epilepsy care. The studies did not discuss pharmacists’ reimbursement for epilepsy care services’ provision. Lastly, the finally included studies were originated by only 3 locations (USA, Netherlands, Palestine), and only 3 search engines.

### Further research

Future research should evaluate pharmacists’ training, alongside evaluating how effective and cost-effective pharmacist-led services are in managing epilepsy, including overall disease management, medicine reconciliation service, patient adherence and concordance, as well as epileptic patients’ education.

Furthermore, pharmacists’ roles are increasing internationally with emphasis on digital health tools and telepharmacy (Babar ZUD, 2023). Therefore, future research could evaluate the delivery of pharmacist-led epilepsy interventions using digital infrastructure (Mantel-Teeuwisse et al., 2021; Menon & Sander, 2021).

## Conclusion

Pharmacists as healthcare professionals within the primary health care setting can have a potential role in running pharmacist-led epilepsy interventions. Pharmacist-led epilepsy interventions are not commonly provided by community pharmacists due to insufficient training. Enhancing the success of service delivery necessitates comprehensive education and regular training for all pharmacists. Furthermore, it is essential to address pharmacists’ time limitations and ensure adequate compensation to support effective service provision.

Finally, it is crucial to increase awareness among both pharmacists and epileptic patients about the potential contributions of pharmacists to improving epilepsy care. Additionally, it is important to ensure that these interventions are consistently offered in community pharmacy settings and/or outpatient clinics.

## Supplementary Material

Supplemental Material S1

Supplemental Material S3

Supplemental Material S2

## Data Availability

All authors had full access to the data that underpin the findings of this study. All data collected are included within the manuscript.

## Data access

Only the authors have full access to the study data, which is secured with a password and stored on servers accessible only to them.
