# Supplementary material for: The role of pharmacists in enhancing epilepsy care: a systematic review of community and outpatient interventions
Source: J Pharm Policy Pract. 2025 Apr 10;18(1):2487046. doi: 10.1080/20523211.2025.2487046 (PMC11986871; doi:10.1080/20523211.2025.2487046)
Supplement: Supplemental Material S3 [file JPPP_A_2487046_SM9261.docx]

**Supplementary Material S3**

**Table 1.** Search Strategy for each database

| **Search Strategy** | **Results** |
| --- | --- |
| **Scopus** | |
| (TITLE-ABS-KEY (epilep*) AND TITLE-ABS-KEY ("community pharmac*")  AND TITLE-ABS-KEY (service* OR intervention* OR implementation* OR consultation* OR care OR compliance OR adherence OR concordance OR morisky OR mars OR educat* OR "quality of life" OR qolie) AND NOT TITLE-ABS-KEY ("hospital pharm*")) | 46 |
| **PubMed** | |
| (((epilep*[Title/Abstract]) AND ("community pharmac*"[Title/Abstract])) AND (service*[Title/Abstract] OR intervention*[Title/Abstract] OR implementation*[Title/Abstract] OR consultation*[Title/Abstract] OR care[Title/Abstract] OR compliance[Title/Abstract] OR adherence[Title/Abstract] OR concordance[Title/Abstract] OR morisky[Title/Abstract] OR mars[Title/Abstract] OR educat*[Title/Abstract] OR "quality of life"[Title/Abstract] OR qolie[Title/Abstract])) NOT ("hospital pharm*"[Title/Abstract]) | 27 |
| **Science Direct** In Science Direct, the limitations of using no more than eight Boolean operators and the inability to employ the truncation operator (*) within a single search query necessitated the creation of four distinct search queries. These queries utilised variations of the same keywords, where applicable, to enhance result retrieval (e.g., using "community pharmacist" instead of "community pharmacy" yielded additional results). In total, ten separate search queries were developed to encompass all potential keyword combinations. However, six of these queries produced entirely duplicate results and were therefore excluded from the synthesis. | |
| Search query No. 1 | |
| Title, abstract or author-specified keywords: epilepsy AND "community pharmacy" AND (service OR intervention OR implementation OR consultation OR care) NOT "hospital pharmacy" | 6 |
| Search query No. 2 | |
| Title, abstract or author-specified keywords: epilepsy AND "community pharmacy" AND (compliance OR adherence OR concordance OR morisky OR mars) NOT "hospital pharmacy" | 4 |
| Search query No. 3 | |
| Title, abstract or author-specified keywords: epilepsy AND "community pharmacy" AND (education OR "quality of life" OR qolie) NOT "hospital pharmacy" | 6 |
| Search query No. 4 | |
| Title, abstract or author-specified keywords: epilepsy AND "community pharmac**ist**" AND (service OR intervention OR implementation OR consultation OR care) NOT "hospital pharmac**ist**" | 11 |

**Table 2.** List of the 46 Scopus results (the remaining 54 results from PubMed and Science Direct were duplicates), inclusion or exclusion status and reason for the exclusion (following the step-by-step exclusion according to PRISMA flowchart).

| **#** | **Title** | **Authors** | **Year** | **DOI** | **Status** | **Exclusion reasoning** |
| --- | --- | --- | --- | --- | --- | --- |
| 4 | Community pharmacists’ knowledge, attitudes toward epilepsy and availability of antiepileptic drugs in Ouagadougou (Burkina Faso) | Dabilgou A.A., et al. | 2023 | 10.1186/s41983-023-00686-8 | Excluded | 1. Identification stage - Based on study population |
| 26 | Pharmacists’ knowledge of issues in pharmacotherapy of epilepsy using antiepileptic drugs: A cross-sectional study in Palestinian pharmacy practice | Shawahna R., et al. | 2017 | 10.1016/j.yebeh.2016.11.027 | Excluded | 1. Identification stage - Based on study population |
| 30 | Access to antiepileptic drug therapy in children in Camagüey Province, Cuba | Arencibia Z.B., et al. | 2012 | 10.1111/j.2042-7174.2012.00215.x | Excluded | 1. Identification stage - Based on study population |
| 31 | Changes in anticonvulsant prescribing for Australian children: Implications for Quality Use of Medicines | Cohen S.A., et al. | 2012 | 10.1111/j.1440-1754.2011.02223.x | Excluded | 1. Identification stage - Based on study population |
| 32 | Knowledge and attitude of iranian community pharmacists about the pharmaceutical care for epileptic females | Sabzghabaee A.M., et al. | 2012 |  | Excluded | 1. Identification stage - Based on study population |
| 3 | Trends in cost and consumption of essential medicines for non-communicable diseases in Azerbaijan, Georgia, and Uzbekistan, from 2019 to 2021 | Kadyrova N., et al. | 2023 | 10.1371/journal.pone.0294680 | Excluded | 1. Identification stage - Based on title - irrelevant to our review |
| 5 | Availability, price, and affordability of antiseizure medicines in Addis Ababa, Ethiopia | Hailemariam F.H., et al. | 2023 | 10.1002/epi4.12792 | Excluded | 1. Identification stage - Based on title - irrelevant to our review |
| 6 | Shortages of antiseizure medications in Australia and the association with patient switching, and adherence in a community setting | Welton J., et al. | 2023 | 10.1016/j.yebeh.2023.109145 | Excluded | 1. Identification stage - Based on title - irrelevant to our review |
| 7 | Trends in antiseizure medications utilization among women of childbearing age with epilepsy in Poland between 2015 and 2019 | Wójcik K., et al. | 2023 | 10.1016/j.yebeh.2023.109091 | Excluded | 1. Identification stage - Based on title - irrelevant to our review |
| 8 | Factors influencing community pharmacists’ knowledge about women’s issues in epilepsy | Jairoun A.A., et al. | 2023 | 10.3389/fpubh.2023.1251393 | Excluded | 1. Identification stage - Based on title - irrelevant to our review |
| 9 | Community pharmacists’ skills and practice regarding dispensing fiscalized substances: a cross-sectional survey | El-Dahiyat F., et al. | 2023 | 10.3389/fphar.2023.1237306 | Excluded | 1. Identification stage - Based on title - irrelevant to our review |
| 12 | Cannabis use in patients with insomnia and sleep disorders: Retrospective chart review | Vaillancourt R., et al. | 2022 | 10.1177/17151635221089617 | Excluded | 1. Identification stage - Based on title - irrelevant to our review |
| 13 | Pet Medications: A Tail of Caution | Nguyen T.T., et al. | 2022 | 10.1177/0897190020966149 | Excluded | 1. Identification stage - Based on title - irrelevant to our review |
| 18 | Medication therapy problems and vaccine needs identified during initial appointment-based medication synchronization visits | Ariyo O., et al. | 2019 | 10.1016/j.japh.2019.04.019 | Excluded | 1. Identification stage - Based on title - irrelevant to our review |
| 19 | Continuing pharmacy education practices in geriatric care among pharmacists in the Upper Midwest | Marvanova M., et al. | 2019 | 10.1016/j.japh.2018.12.020 | Excluded | 1. Identification stage - Based on title - irrelevant to our review |
| 20 | Assessment of potentially inappropriate medications in elderly according to Beers 2015 and STOPP criteria and their association with treatment satisfaction | Sakr S., et al. | 2018 | 10.1016/j.archger.2018.06.009 | Excluded | 1. Identification stage - Based on title - irrelevant to our review |
| 22 | Pregabalin Misuse and Abuse in Jordan: a Qualitative Study of User Experiences | Al-Husseini A., et al. | 2018 | 10.1007/s11469-017-9813-4 | Excluded | 1. Identification stage - Based on title - irrelevant to our review |
| 23 | Pregabalin dispensing patterns in Amman-Jordan: An observational study from community pharmacies | Al-Husseini A., et al. | 2018 | 10.1016/j.jsps.2018.01.012 | Excluded | 1. Identification stage - Based on title - irrelevant to our review |
| 27 | Evaluation of the Most Frequently Prescribed Extemporaneously Compounded Veterinary Medications at a Large Independent Community Pharmacy | Karara A.H., et al. | 2016 |  | Excluded | 1. Identification stage - Based on title - irrelevant to our review |
| 33 | Pharmacogenomics in a community pharmacy: ACT now | Padgett L., et al. | 2011 | 10.1331/JAPhA.2011.10178 | Excluded | 1. Identification stage - Based on title - irrelevant to our review |
| 35 | Patients with epilepsy's perception on community pharmacist's current and potential role in their care | McAuley J.W., et al. | 2009 | 10.1016/j.yebeh.2008.10.014 | Excluded | 1. Identification stage - Based on title - irrelevant to our review |
| 36 | An assessment of patient and pharmacist knowledge of and attitudes toward reporting adverse drug events due to formulation switching in patients with epilepsy | McAuley J.W., et al. | 2009 | 10.1016/j.yebeh.2008.08.009 | Excluded | 1. Identification stage - Based on title - irrelevant to our review |
| 38 | Quality of antiepileptic drugs in Vietnam | Mac T.L., et al. | 2008 | 10.1016/j.eplepsyres.2008.03.006 | Excluded | 1. Identification stage - Based on title - irrelevant to our review |
| 40 | Revealing undetected problems with medication therapy management services | Alexander A.J., et al. | 2008 | 10.4140/TCP.n.2008.473 | Excluded | 1. Identification stage - Based on title - irrelevant to our review |
| 43 | Recruitment of a cohort of lamotrigine users through community pharmacists: Differences between patients who gave informed consent and those who did not | Knoester P.D., et al. | 2005 | 10.1002/pds.992 | Excluded | 1. Identification stage - Based on title - irrelevant to our review |
| 44 | Patterns of lamotrigine use in daily clinical practice during the first 5 years after introduction in the Netherlands | Knoester P.D., et al. | 2004 | 10.1111/j.1365-2710.2004.00544.x | Excluded | 1. Identification stage - Based on title - irrelevant to our review |
| **Table 2. (continued)** | | | | | | |
| **#** | **Title** | **Authors** | **Year** | **DOI** | **Status** | **Exclusion reasoning** |
| 46 | General medical practitioners' attitudes towards the use of patient medication records | Rogers P.J., et al. | 1995 | 10.1111/j.2042-7174.1995.tb00811.x | Excluded | 1. Identification stage - Based on title - irrelevant to our review |
| 37 | Ensuring access to psychotropic medication in sub-Saharan Africa | Eaton J. | 2008 | 10.4314/ajpsy.v11i3.30227 | Excluded | 1. Identification stage - Based on title - irrelevant to our review |
| 41 | Malaria chemoprophylaxis advice: Survey of South African community pharmacists' knowledge and practices | Toovey S. | 2006 | 10.1111/j.1708-8305.2006.00035.x | Excluded | 1. Identification stage - Based on title - irrelevant to our review |
| 15 | Effects of the COVID-19 pandemic on medication adherence: In the case of antiseizure medications, A scoping review | Menon S., et al. | 2021 | 10.1016/j.seizure.2021.10.009 | Excluded | 1. Identification stage - Based on title - Review article |
| 17 | Community pharmacists’ role in caring for people living with epilepsy: A scoping review | Bacci J.L., et al. | 2021 | 10.1016/j.yebeh.2021.107850 | Excluded | 1. Identification stage - Based on title - Review article |
| 34 | The quality of private pharmacy services in low and middle-income countries: A systematic review | Smith F. | 2009 | 10.1007/s11096-009-9294-z | Excluded | 1. Identification stage - Based on title - Review article |
| 24 | Factors limiting adherence to antiepileptic treatment: A French online patient survey | Laville F., et al. | 2018 | 10.1111/jcpt.12615 | Excluded | 2. Screening phase 1 - Based on abstract - 21% of the study's population was <15 years old |
| 10 | Community Pharmacists’ Role in the Treatment of Epilepsy | Rice K., et al. | 2023 |  | Excluded | 2. Screening phase 1 - Based on abstract - irrelevant to our review |
| 21 | A survey on awareness, knowledge, and attitudes toward epilepsy in an urban community in Turkey | Macit C., et al. | 2018 | 10.4103/njcp.njcp_199_17 | Excluded | 2. Screening phase 1 - Based on abstract - irrelevant to our review |
| 42 | AEDs availability and professional practices in delivery outlets in a city center in Southern Vietnam | Mac T.L., et al. | 2006 | 10.1111/j.1528-1167.2006.00425.x | Excluded | 2. Screening phase 1 - Based on abstract - irrelevant to our review |
| 45 | Assessing the needs of pharmacists and physicians in caring for patients with epilepsy. | McAuley J.W., et al. | 1999 | 10.1016/S1086-5802(16)30468-5 | Excluded | 2. Screening phase 1 - Based on abstract - irrelevant to our review |
| 1 | Opportunities for Community Pharmacists to Counsel Patients With Epilepsy and Seizure Clusters to Overcome Barriers and Foster Appropriate Treatment | Gidal B., et al. | 2024 (first published online on 04/10/22) | 10.1177/08971900221126570 | Excluded | 2. Screening phase 1 - Based on abstract - Review article |
| 16 | Medication adherence, utilization of healthcare services, and mortality of patients with epilepsy on opiate replacement therapy: A retrospective cohort study | Askarieh A., et al. | 2021 | 10.1016/j.yebeh.2021.107829 | Excluded | 2. Screening phase 1 - Based on abstract - very specific population |
| 11 | Epilepsy: management | Thomas T. | 2022 | 10.1211/PJ.2022.1.140544 | Excluded | 3. Screening phase 2 - Did not meet the inclusion criteria set |
| 39 | Importance of community pharmacy support for patients with epilepsy | Lumb J. | 2008 |  | Excluded | 3. Screening phase 2 - Review article |
| 2 | Using design thinking to strengthen the community pharmacist's role in epilepsy care | Zaraa S., et al. | 2024 (available online on 29/11/23) | 10.1016/j.yebeh.2023.109542 | Included | N/A |
| 14 | Stakeholder perceptions of community pharmacist population health management of people living with epilepsy | Bacci J.L.,et al. | 2021 | 10.1016/j.yebeh.2021.108389 | Included | N/A |
| 25 | Which information on women's issues in epilepsy does a community pharmacist need to know? A Delphi consensus study | Shawahna R. | 2017 | 10.1016/j.yebeh.2017.09.026 | Included | N/A |
| 28 | Anti-epileptic drug changes and quality of life in the community | Wassenaar M., et al. | 2016 | 10.1111/ane.12478 | Included | N/A |
| 29 | An evaluation of the impact of memory and mood on antiepileptic drug adherence | McAuley J.W., et al. | 2015 | 10.1016/j.yebeh.2014.11.017 | Included | N/A |

* The article number (#) is the same as the one that appeared on the Scopus results page, which displayed the most recent results first.
