# Supplementary material for: The role of pharmacists in enhancing epilepsy care: a systematic review of community and outpatient interventions
Source: J Pharm Policy Pract. 2025 Apr 10;18(1):2487046. doi: 10.1080/20523211.2025.2487046 (PMC11986871; doi:10.1080/20523211.2025.2487046)
Supplement: Supplemental Material S2 [file JPPP_A_2487046_SM9260.docx]

**Supplementary Material S2**

**STUDY PROTOCOL**

**Title: The Role of Pharmacists in Enhancing Epilepsy Care: A Systematic Review of Community and Outpatient Interventions.**

**Review question**

1. What are the current published practices that pharmacists apply to effectively manage patients with epilepsy in a community pharmacy setting or in an outpatient clinic?
2. What types of pharmacist-led interventions are available in a community pharmacy and/or outpatient clinic setting to manage diagnosed patients with epilepsy, educate and train them, monitor and follow-up on medication dosage regimens, and manage high-risk patients?
3. Are there any studies or practices that demonstrate a collaborative approach involving pharmacists for the management of epilepsy?

# Searches

We will use the following electronic databases: Scopus, Pubmed Central and Science Direct. Databased will be searched from their inception until 31/12/2023. We will use keywords and medical subject headings where available to identify studies regarding pharmacists’ potential roles in the management of patients with epilepsy. Boolean operators (AND, OR, NOT) will be used.

# Types of studies to be included

Published studies that answer to the above-mentioned review questions will be included. They may apply different study designs, such as 1. Qualitative, 2. Quantitative randomised controlled trials, 3. Quantitative non-randomised, 4. Quantitative descriptive, 5. Mixed methods.

We will exclude review studies, studies conducted in hospital pharmacy settings, and studies involving children.

# Condition or domain being studied

Epilepsy management.

# Intervention(s), exposure(s)

Studies to include pharmacists’ practices-interventions in epilepsy management.

# Comparator(s)/control

None.

# Context

Community pharmacy settings and/or outpatient clinics, if a pharmacist is involved.

# Main outcome(s)

The scope of the studies included (based on the quality criteria of the MMAT 2018 assessment tool) encompasses: pharmacists’ interventions or activities described for managing patients with epilepsy; the community pharmacy setting and/or outpatient clinics; patient populations; and multidisciplinary collaboration for effective management of epilepsy.

## *Measures of effect*

Not applicable.

# Additional outcome(s)

None.

## *Measures of effect*

Not applicable.

# Data extraction (selection and coding)

This review will be reported in accordance with PRISMA 2020 guideline. The titles and abstracts will be screened to identify potentially eligible articles. All identified articles will be checked against eligibility criteria.

The assessors will review the search results to identify eligible articles and extract the relevant data. Any discrepancies or disagreements will be resolved through additional discussion. The assessors will also screen the full texts to select articles for data extraction, which will be conducted using a Microsoft Excel® spreadsheet.

Extracted data will include author(s), year of publication, location of published article, main objectives of the articles, methods/tools/measurements, and key findings.

# Risk of bias (quality) assessment

Articles included in this review will be assessed by using the English version of the MMAT tool (Mixed Methods Appraisal Tool). It includes 25 questions grouped into 5 categories depending on the different study designs, plus 2 screening questions to be answered in all study designs:
1. Qualitative, 2. Quantitative randomised controlled trials, 3. Quantitative non-randomised,
4. Quantitative descriptive, 5. Mixed methods. For each of the 27 questions the possible answers were three: “Yes”, “No”, “Can’t tell”.

# Strategy for data synthesis

The data will be summarised and presented using summary tables and descriptive paragraphs.

# Analysis of subgroups or subsets

Not applicable.

# Review team members and their organisational affiliations

All research committee members (listed in the manuscript).

# Type and method of review

Systematic review.
